# Molecular Mechanism of Caulis Spatholobi in the Treatment of Chronic Myeloid Leukemia based on Network Pharmacology and Experimental Verification

**DOI:** 10.2174/1573409919666230417085106

**Published:** 2023-09-22

**Authors:** Yanchun Wu, Fangfang Lian, Hongxia Chen, Chaoyu Zhang, Linli Wei, Hui Tian

**Affiliations:** 1 School of Pharmacy, Guangxi University of Chinese Medicine, Guangxi, 530200, China;; 2 The First Affiliated Hospital of Guangxi Medical University, Guangxi, 530021, China

**Keywords:** Caulis spatholobi, chronic myeloid leukemia, mechanism of action, network pharmacology, cell experiment, caspase-3

## Abstract

**Background:**

Caulis Spatholobi is one of the necessary Chinese herbal medicines for hematologists in the treatment of malignant tumors, but its potential targets and molecular mechanisms need further exploration.

**Objective:**

This study aimed to predict the relevant targets of the treatment of chronic myeloid leukemia (CML) with Caulis Spatholobi by applying the network pharmacology method, and *in vitro* cell experiments were conducted to verify the mechanism of Caulis Spatholobi in the treatment of CML.

**Methods:**

TCMSP, ETCM, Genecards, and GisGeNET databases were used to obtain relevant targets of Caulis Spatholobi in the treatment of CML. Go and KEGG analyses were performed using the David database. Using Cytoscape 3.7.2, the “active compounds-targets-pathways” network was constructed. Further validation was carried out by pharmacological experiments *in vitro*. The proliferation and apoptosis of K562 cells were observed by the MTT method and Hoechst 33242 fluorescence staining method. The predicted targets and their related signal pathways were verified by western blotting.

**Results:**

In this study, 18 active compounds and 43 potential targets were obtained. The results of the MTT method showed that compared with the normal control group, 62.5-500 μg/mL alcohol extract of Caulis Spatholobi had an obvious inhibitory effect on K562 and the IC_50_ value was less than 100 μg/mL. The Hoechst 33242 fluorescence staining method showed that the alcohol extract of Caulis Spatholobi could promote apoptosis. The results of western blotting showed that compared with the normal control group, the expressions of Bax and Caspase-3 proteins in the 62.5 and 125 μg/mL alcohol extract of Caulis Spatholobi groups were significantly up-regulated (*p* < 0.05). The expression of Bcl-2 in the 125 μg/mL alcohol extract of the Caulis Spatholobi group was significantly down-regulated (*p* < 0.01), and the expression of Bcl-2 in the 62.5 and 31.25 μg/mL alcohol extract of Caulis Spatholobi groups was also significantly down-regulated (*p* < 0.05). It showed that the ethanol extract of Caulis Spatholobus could promote apoptosis by up-regulating the expression of Bax and caspase-3 and down-regulating the expression of the Bcl-2 protein.

**Conclusion:**

The treatment of Caulis Spatholobi for CML has the characteristics of multi-targets and multi-pathways. The results of *in vitro* pharmacological experiments demonstrated that its mechanism of action might be based on the expression of key target proteins, such as Caspase-3, Bcl-2, and Bax, thereby inhibiting cell proliferation and promoting cell apoptosis, which provides a scientific basis for the treatment of CML.

## 1 INTRODUCTION

CML is a malignant tumor of the hematopoietic system. It is a disease derived from clonal abnormalities of hematopoietic stem cells and specific Ph chromosomes or BCR/ABL fusion genes [1, 2]. From 1990 to 2017, the incidence rate of leukemia in China increased significantly. The incidence rate of leukemia in China has risen the highest in the past 28 years [3]. CML is a subtype of leukemia, accounting for approximately 20% of adult leukemias [4]. The commonly used western medicine treatment methods include tyrosine kinase inhibitor (TKI), chemotherapy and immunomodulatory drugs. Although they can effectively reduce mortality and prolong survival time, they are difficult to cure fundamentally. However, long-term use will lead to adverse drug reactions and drug resistance, and the patients will bear great economic pressure. Chinese medicine treatment of CML, while obtaining the exact curative effect because Chinese medicine is cheaper than western medicine, can greatly reduce the economic burden of patients and their families, can improve the quality of life of patients, and has the advantages of safety, less side effects, *etc*.

Caulis Spatholobi is the dried vine stem of *Spatholobus suberectus* Dunn, a commonly used traditional Chinese medicine that grows in the deep mountains of Guangxi and Yunnan. The cross-section of Caulis Spatholobi exudes a red resinous substance. In traditional Chinese medicine, it is considered to have the effect of promoting blood circulation, nourishing blood, clearing the meridians, and activating collaterals. Caulis Spatholobi is one of the necessary Chinese herbal medicines for hematologists in the treatment of malignant tumors. It was found that in Guangxi, ancient people used Caulis Spatholobi or “Xue Teng” plants with red resin to treat leukemia, lymphoma and other tumor diseases. There are literature reports on the commonly used traditional Chinese medicine compounds and methods of use in the clinical treatment of leukemia. Caulis Spatholobi is one of the traditional Chinese medicines commonly used in these compounds [5-7]. At the same time, modern pharmacological studies have shown that Caulis Spatholobi has inhibitory effects on acute monocytic leukemia cell lines U937, MCF-7 cells, mouse breast cancer 4T1 cells, human fibrosarcoma HT1080, and other tumor cells, and its mechanism of action is mainly related to inducing tumor cell apoptosis, arresting cell cycle, inhibiting tumor cell metastasis, and scavenging free radicals [8-10]. A great deal of data is available on the use of Caulis Spatholobi in the treatment of cancer, especially blood cancer. It has been reported in the literature that Caulis Spatholobi can inhibit the proliferation of mouse lymphocytic leukemia cell L1210 and the induced transplanted tumor [11], but the mechanism of action is not comprehensive.

Network pharmacology is a new discipline based on the theory of systems biology, which analyzes the network of biological systems and selects specific signaling nodes for multi-target drug molecular design. Currently, it has become an indispensable method for exploring the underlying mechanism of traditional Chinese medicine [12]. This method is mainly used for many traditional Chinese medicines and compound prescriptions to reveal the complex biological network relationship among drugs, components, targets, and diseases.

Therefore, this study used the method of network pharmacology to construct the network of active ingredients-targets-pathways and systematically explore the multi-pathway regulation of Caulis Spatholobi. Further experimental verification was carried out in combination with the pharmacological experiment *in vitro*. The effect of the alcohol extract of Caulis Spatholobi on the proliferation of K562 cells was explored through the MTT experiment. The apoptosis of K562 cells was observed by Hoechst 33242 fluorescence staining. The predicted targets were verified by Western blotting in order to clarify the mechanism of Caulis Spatholobi in the treatment of CML, thus providing ideas for the basic research on Caulis Spatholobi and then laying a foundation for clinical research.

## 2 MATERIALS

Caulis Spatholobi was purchased from Guangxi Xianzhu Traditional Chinese Medicine Technology Co., Ltd. and was identified by professor Wenfang Ma of the Guangxi University of Chinese medicine. K562 cell lines were purchased from KeyGEN BioTECH. The source of K562 cells was bone marrow. 96-well cell culture plates, 24-cell culture plates, and cell culture flasks were purchased from Corning Company in the United States. Fetal bovine serum was purchased from Hangzhou Sijiqing Company, and DMEM culture medium was purchased from Gibco Company in the United States. MTT and DMSO were purchased from Beijing SoLarbio Technology Co., Ltd., and Hoechst 33242 dye was purchased from KGI Biotechnology Development Co., Ltd. Caspase-3, BAX, and Bcl-2 antibodies were purchased from cell signaling. The BCA protein content kit was purchased from Nanjing Jiancheng Bioengineering Institute. Bcl-2 (D17C4) Rabbit mAb (338 μg/ml, 1:1000, #3498) and Caspase-3 (D3R6Y) Rabbit mAb (280 μg/ml, 1:1000, #14220) were purchased from Cell Signaling Technology. Bax monoclonal antibody (1600 μg/ml, 1:1000, CloneNo.: 4G5E8) was purchased from Proteintech Group, Inc. β-actin (4D3) monoclonal antibody (1:10000, BS6007M) was purchased from Bioworld.

## 3 METHODS

### 3.1 Active Ingredients and Relevant Targets Screening of Caulis Spatholobi

Through the pharmacology database and analysis platform of the traditional Chinese medicine system (https://tc mspw.com/tcmsp.php) and Encyclopedia of traditional Chinese medicine database (http://www.tcmip.cn/ETCM/index.php/Home/Index/) chemical constituents of Caulis Spatholobi were searched [13, 14]. All chemical composition information through the PubChem database (https://pubchem.n cbi.nlm.nih.gov/) was downloaded in the “2dsdf” structure file. The “2dsdf” structure files of each component were copied and input into the SwissADME database (http://www.swissadme.ch/) for screening the potential core active ingredients. The criteria are as follows: ① GI absorption value is “high,” indicating that the oral bioavailability of the ingredient is good; ② two or more of the results of the five attributes (Lipinski, Ghose, Veber, Egan, Muegge) in the drug-likeness column were positive. The screened compounds were predicted by the TCMSP database and Swiss target prediction database (http://www.swisstargetprediction.ch/) to obtain their corresponding targets. All targets were standardized in the UniProt protein database (https://www.unip rot.org).

### 3.2 Screening for Disease Targets

“Chronic myeloid leukemia” was used as the keyword to obtain potential targets for the treatment of CML in the Genecards and DisGeNET databases. After merging two disease database targets, the duplicate value was deleted to get the CML targets.

### 3.3 PPI Network Construction

In order to clarify the interaction between the active component targets of Caulis Spatholobi and CML targets, the data of the two targets were imported into the Venny 2.1 database to obtain intersect targets and draw Venn diagrams. Then, the intersection targets were submitted to the STRING11.0 database to construct a protein-protein interaction (PPI) network model. The biological species was set as “*Homo sapiens*”, and the minimum interaction threshold was set as “medium confidence” > 0.9. The rest were default settings. The non-interacting targets were removed, and a PPI network of intersection targets was constructed for the treatment of CML by Caulis Spatholobi.

### 3.4 Go Biological Process and KEGG Pathway Enrichment Analysis

The targets of the treatment of CML by Caulis Spatholobi were entered into the DADIV database, and *p* < 0.01 was set to analyze its main biological processes and metabolic pathways and conduct an enrichment analysis. The data results were saved, and online drawing tools were used to visualize the data.

### 3.5 Construction of an Active Components-targets-pathway Network of Caulis Spatholobi

CytoScape 3.7.2 was used to construct the active ingredient-intersection target-pathway network of Caulis Spatholobi. The built-in tools of CytoScape 3.7.2 were used to analyze the network topology parameters of active ingredients and targets, including degree, betweenness, closeness, *etc*. The network topology parameters were employed to determine the core targets and the main active components that exert drug efficacy.

### 3.6 *In vitro* Validation Experiment

#### 3.6.1 Effects of Different Concentrations of Alcohol Extract of Caulis Spatholobi on Cell Viability

1 × 10^4^ cells were plated onto 96-well culture plates and treated with various concentrations of the alcohol extract of Caulis Spatholobi (0,15.625, 31.25, 62.5, 125, 250, and 500 μg/mL) in fresh medium for 48 h. The medium was aspirated, and MTT (50 μL, 5 mg/mL in phosphate-buffered saline (PBS)) was added to each well. Incubation was continued for an additional 4 h at 37°C. The supernatant was discarded; DMSO of 100 ml to each well was added, shaken, and mixed well, and then the OD value was measured with a wavelength of 570 nm using the microplate reader. The calculation was done by using the following equation:

Cell proliferation inhibition rate (%) = [1-(OD_570_ of experimental group/OD_570_ of the blank control group)]×100%.

According to the results of this experiment, three appropriate concentrations were selected for subsequent experiments.

#### 3.6.2 Observation of Apoptosis by Hoechst 33242 Staining

1×10^4^ cells were plated onto 96-well culture plates and treated with various concentrations of the alcohol extract of Caulis Spatholobi (0, 31.25, 62.5, and 125 μg/mL) in fresh medium for 48 h. They were then continued to culture in a CO_2_ incubator for 48 hours, and K562 cells were cultured for 48 hours and centrifuged at 1200 rpm. Then, the supernatant was absorbed, and cells were washed with PBS once. The fixed solution was added to each well. They were then placed at 4°C for 60 minutes, and the solution was then discarded. After washing with PBS, Hoechst 33242 staining solution was added and incubated at 37°C for 30 min under a fluorescence microscope to observe and take photos.

#### 3.6.3 The Protein Expression Levels of Caspase-3, Bax, and Bcl-2 Determined by Western Blot

The K562 cells cultured for 48 hours were centrifuged at 12000 rpm for 5 min. The supernatant was washed with PBS twice, and the protein content was determined by the BCA method. The concentration was quantified to 2 μg/μL, and then 4 × protein-loaded buffer was added. It was then put into 100°C hot water to inactivate for 10 minutes. The protein lysates were separated by electrophoresis in 12% SDS polyacrylamide gel and blotted onto a nitrocellulose membrane. Proteins were detected using monoclonal antibodies and visualized using anti-rabbit IgG conjugated with peroxidase (HRP) as the HRP substrate.

#### 3.6.4 Data Processing

The data are expressed as X ± sd. Statistical comparisons were made by t-test. *p* < 0.05 was considered significant.

## 4 RESULTS

### 4.1 Network Pharmacology of Caulis Spatholobi for the Treatment of CML

#### 4.1.1 Acquisition of Active Ingredient Targets in Caulis Spatholobi

We explored the potential targets of 63 potential pharmacological active ingredients and obtained 321 targets.

#### 4.1.2 Acquisition of CML-relevant Targets

The disease targets of Genecards and TDD databases were merged, and duplicate values were removed. Finally, 8677 CML-related targets were obtained.

#### 4.1.3 PPI Network Construction

**Fig. (1):**
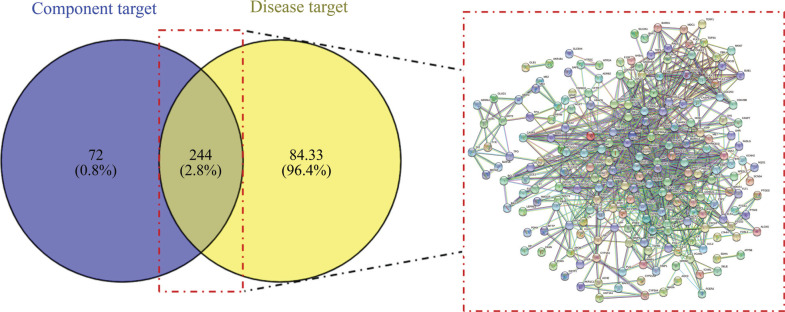
Venn diagram and PPI network diagram.

Taking the intersection of the active component targets of Caulis Spatholobi and the CML targets, 244 common targets were obtained, that is, the potential targets of Caulis Spatholobi in the treatment of CML, and they were drawn into a Venn diagram. Then, the intersection targets were submitted to the STRING 11.0 platform to obtain the PPI network (Fig. **1**).

#### 4.1.4 Go Biological Process and KEGG Pathway Enrichment Analysis

**Fig. (2):**
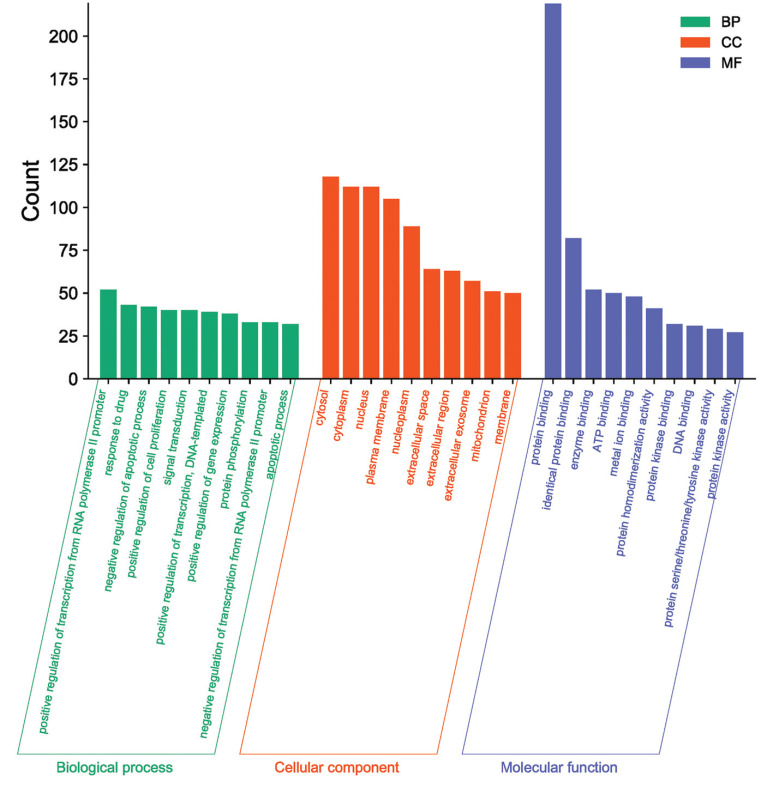
GO enrichment analysis results.

A total of 244 common targets were analyzed through the David database. The top 10 results of the GO biological process were screened according to the order of *p* value from small to large (Fig. **2**). It includes positive and negative RNA polymerase II promoter transcription regulation, signaling transduction, negative regulation of apoptosis, positive regulation of cell proliferation, positive regulation of transcription, cell proliferation, positive regulation of gene expression, transcription factor binding, and other processes. The results indicated that Caulis Spatholobi was involved in various mechanisms, such as the regulation of apoptosis, gene expression, and cell metabolism in the treatment of CML.

**Fig. (3):**
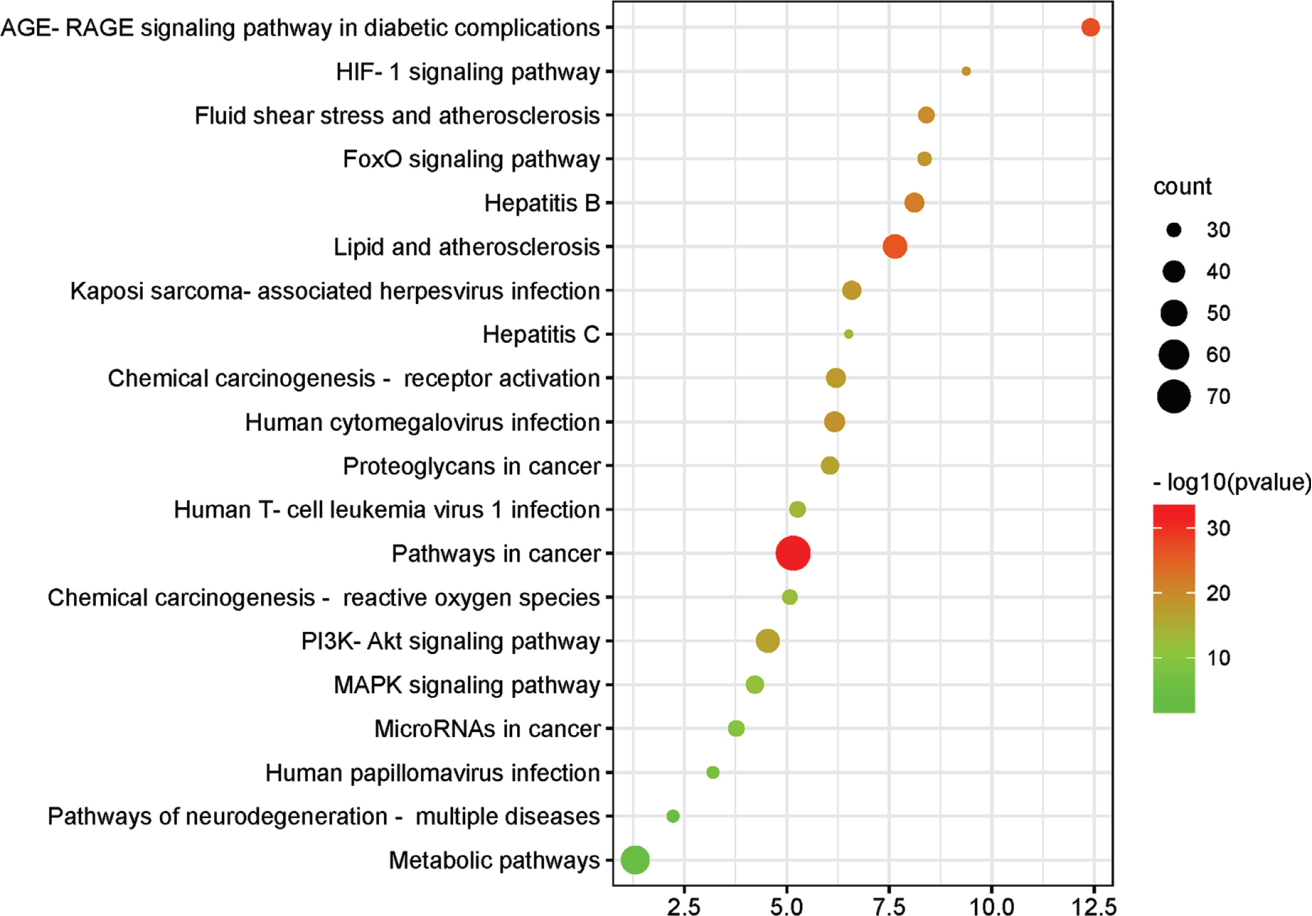
KEGG enrichment analysis results: The color of the bubbles from red to green represents the *p* value from small to large. The smaller the *p* value, the stronger the significance. A larger size of a bubble means a greater gene count.

In order to reveal the related pathways of CaulisSpatholobi in the treatment of CML, 224 key targets were analyzed by KEGG through the David database, and 189 related pathways of Caulis Spatholobi in the treatment of CML were obtained. This includes pathways in cancer, metabolic pathways, lipid and atherosclerosis, PI3K-Akt signaling pathway, human cytomegalovirus infection, hepatitis B, chemical carcinogenesis, receptor activation, etc. We screened the first 20 pathways and drew a KEGG bubble diagram (Fig. **3**).

#### 4.1.5 Construction of Active Components-targets-pathway Network of Caulis Spatholobi

**Fig. (4):**
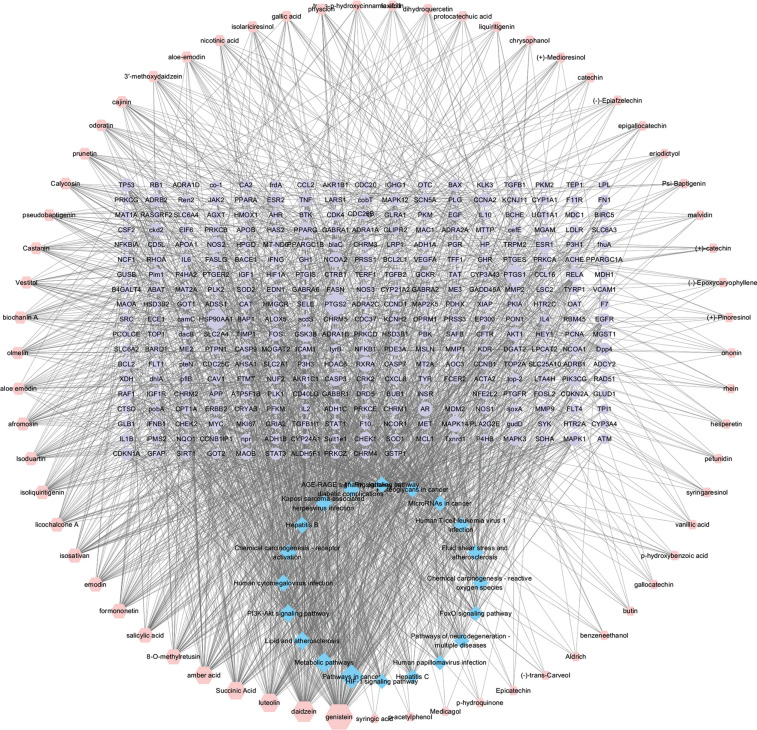
Caulis Spatholobi active ingredient-key target-pathway: the red circle node represents the potential active ingredient in the network. Blue circle nodes represent pathways, and purple circle nodes represent targets. A larger size of a node means a greater degree.

The active components, intersection targets, and pathway data of Caulis Spatholobi were imported into Cytoscape 3 7.2 software, which was used to visually analyze the above networks. The topology parameters in the network were analyzed through the network analyzer tool. Then, the size of the node shape was adjusted according to the degree value (Fig. **4**). Taking the median values of “Degree”, “Closeness Centrality”, and “Better Centrality” as the initial conditions, we successively screened the median value nodes of ≥ “Degree” and obtained 18 compounds as potential bioactive components (Table **1**) and 43 action targets in this study (Table **2**). Genistein, daidzein, formononetin, emodin, isoliquiritigenin, aloe emodin, and gallic acid were all identified in the ethanol extract [15-19]. We selected the ethanol extract of Caulis Spatholobi for further experimental verification.

### 4.2 Experimental Validation in Cells

#### 4.2.1 The Effect of Alcohol Extracts of Caulis Spatholobi on Cell Proliferation

Celastrol extract inhibited the growth of K562 cells compared to the control in a concentration-dependent manner. Cell viability decreased with increasing concentrations of the extract of the alcohol extracts of Caulis Spatholobi. It exerted an obvious inhibitory effect at the concentration of 62.5-500 μg/mL. The IC_50_ value was found to be less than 100 μg/mL (Table **3**).

#### 4.2.2 Morphological Observations of Apoptosis in Each Group

The morphology of the cells in the blank control group was round, with well-defined borders, abundantcytoplasm and nuclei showing diffuse and uniform fluorescence. After the administration of drugs, the morphology of the cells changed significantly and dense blue granules containing bulk fluorescence in the cytoplasm or nucleus were observed, along with irregular and strongly fluorescent cell debris and spherical protrusions, as shown in Fig. (**5**).

#### 4.2.3 The Protein Level Expression of Caspase-3, Bax and Bcl-2 in Each Group was Determined by Western Blot

The results are shown in Fig. (**6**). Compared with the normal control group, the expressions of Bax and Caspase-3 proteins in the 62.5 and 125 μg/mL Caulis Spatholobialcohol extracts groups were significantly up-regulated(*p* < 0.05). It was reported that 125 μg/mL of chick sanguinarine extract caused a significant downregulation of Bcl-2 expression (*p* < 0.01). The expression of Bcl-2 in the alcohol extracts groups of 62.5 and 31.25 μg/mL Caulis Spatholobi was significantly down-regulated (*p* < 0.05). The proportion of Bcl-2/Bax in the alcohol extracts groups of 125 and 62.5 μg/mL Caulis Spatholobi was significantly down-regulated (*p* < 0.05). It indicated that the alcohol extracts of Caulis Spatholobi could inhibit cell apoptosis by up-regulating Bax and Caspase-3 and down-regulating Bcl-2 protein expression. This also suggests that the up-regulation of the Bax/Bcl-2 ratio leads to the release of cytochrome C in the mitochondrial membrane, activates the expression of caspase-3 protein and mitochondrial apoptosis pathway, and leads to the apoptosis of K562 cells.

## 5 DISCUSSION

CML is a malignant myeloproliferative tumor originating from hematopoietic stem cells, and the BCR-ABL fusion protein with tyrosine kinase activity is the core factor leading to the disease.

Apoptosis is the programmed death of cells in the body. It plays an important role in the maintenance of the internal environment and the development of multiple systems. Apoptosis is strictly regulated by the Bcl-2 family, caspase family and other genes. Drugs usually play an anti-tumor effect through apoptosis, inhibition of cell proliferation, cycle arrest, and metastasis. The mechanism of apoptosis is complex, involving many apoptosis factors. In this study, 43 key targets of Caulis Spatholobi for the treatment of CML were obtained. In order to explore the mechanism of Caulis Spatholobi in the treatment of chronic myeloid leukemia, we selected Bcl-2, Bax, caspase-3, and other classical proteins that play an important role in cell apoptosis for further experimental verification. Tu *et al.* found that indomethacin can inhibit the proliferation of chronic myeloid leukemia cells and down-regulate Bcl-2 gene expression to induce apoptosis [20]. Bax is the homologous gene of Bcl-2, which is mainly located in the cytoplasm. When stimulated by apoptosis signals, it migrates from the cytoplasm to the outer mitochondrial membrane, undergoes conformational changes and oligomerization, and mediates the release of downstream apoptosis molecules, thereby triggering apoptosis [21]. After the apoptosis program is initiated, the mitochondrial pathway, death receptor pathway, and endoplasmic reticulum stress lead to the activation of Caspase-12 and finally, the activation of Caspase-3, which leads to the occurrence of apoptosis [22]. Caspase-3 is a common downstream effector part of multiple apoptosis pathways and occupies a central position in the process of apoptosis. Known as the “death-executing protease”, it has been used in research on the proliferation and apoptosis of various tumor cells, with a mature technical route and high stability [23].

Most of the network pharmacology research data is based on published experimental studies. The number of drug-target effects that have been experimentally verified is limited and cannot reveal the complete mechanism of action of drugs in treating diseases. The construction of theoretical models requires experiments to verify their scientificity, and the same is true for network pharmacology research. The accuracy and reliability of the prediction results can only be verified by combining the prediction results of network pharmacology with experimental verification [24]. Therefore, in the KEGG pathway enrichment analysis, we selected the highly enriched PI3K-AKT signaling pathway for *in vitro* validation experiments and carried out downstream regulated apoptosis-related targets Bcl-2, Bax, and Caspase-3. Further experiments were carried out to verify and observe the staining of K562 cell apoptosis in each drug group, aiming to verify that Caulis Spatholobi may interfere with CML through PI3K-AKT and other signaling pathways. Three drug concentrations of 32.15, 62.5, and 125 μg/mL of Caulis Spatholobi alcohol extracts were screened by MTT experiment. Our results showed that compared with the blank control group, the alcohol extracts of Caulis Spatholobi could effectively inhibit the proliferation of K562 cells and induce cells by up-regulating the expression levels of apoptotic proteins Caspase-3 and Bax and down-regulating the expression levels of anti-apoptotic proteins Bcl-2. It was reported that Caulis Spatholobi can down-regulate the PI3K-AKT signaling pathway, and hence, we speculated that induction of apoptosis of K562 cells by regulating the PI3K-AKT pathway might be one of the main mechanisms of Caulis Spatholobi in the treatment of CML. This further verifies the scientificity and reliability of network pharmacology in predicting that drugs act on disease targets.

## CONCLUSION

In this study, the mechanism of Caulis Spatholobi treatment of CML was systematically analyzed by network pharmacology and *in vitro* pharmacological validation experiments, indicating that Caulis Spatholobi treatment of CML plays a synergistic role through multiple components, multiple targets, and multiple pathways. The mechanism may involve affecting the expression of key target proteins, such as Caspase-3, Bcl-2 and Bax, thus inhibiting cell proliferation and promoting cell apoptosis. This study provides the direction for further pharmacodynamic substance basis and mechanism of action. Moreover, it verified related targets of Caulis Spatholobi in CML treatment and the inhibitory effect of K562 cells *in vitro* through *in vitro* pharmacological experiments, thus providing relevant ideas for preclinical research.

## Figures and Tables

**Fig. (5):**
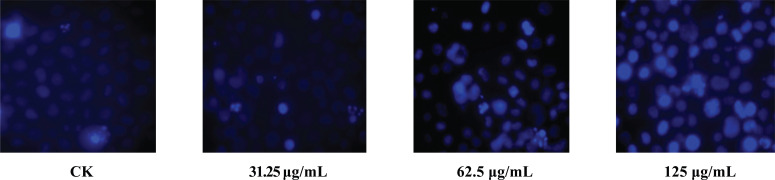
Fluorescence plots of apoptosis in each group (× 200).

**Fig. (6):**
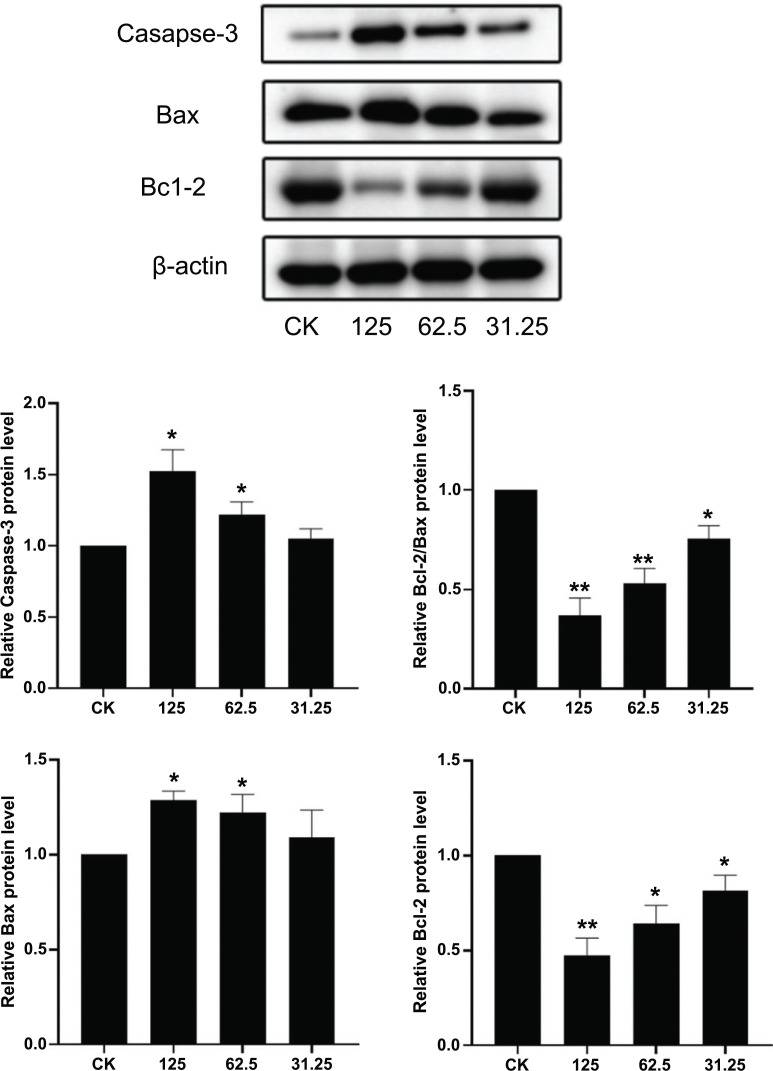
Protein levels of Caspase-3, Bax and Bcl-2 in each group. (Compared with the normal control *p < 0.05, **p < 0.01.

**Table 1:** Active ingredients of Caulis Spatholobi in the treatment of chronic myeloid leukemia.

**Number**	**Mol ID**	**Molecule Name**	**Betweenness Centrality**	**Closeness Centrality**	**Degree**
1	MOL000481	Genistein	0.19091557	0.42088608	95
2	MOL000390	Daidzein	0.12849555	0.399	68
3	MOL000006	Luteolin	0.0651506	0.39117647	56
4	MOL003635	8-O-methylretusin	0.04411745	0.36405109	39
5	MOL001801	Salicylic acid	0.07305431	0.37712665	36
6	MOL000392	Formononetin	0.02085598	0.35816876	36
7	MOL000472	Emodin	0.03016421	0.37570621	33
8	MOL000509	Isosativan	0.02551005	0.3568873	33
9	MOL000497	Licochalcone A	0.01889274	0.35625	31
10	MOL001789	Isoliquiritigenin	0.01896083	0.35498221	30
11	MOL000467	Afromosin	0.01093663	0.35309735	27
12	MOL002985	Isoduartin	0.01012541	0.35309735	27
13	MOL000471	Aloe emodin	0.0124286	0.36740331	23
14	MOL000500	Vestitol	0.00780182	0.35	22
15	MOL000467	Castanin	0.00501979	0.34877622	21
16	MOL000471	Aloe-emodin	0.00642106	0.36206897	16
17	MOL000421	Nicotinic acid	0.03096237	0.35816876	16
18	MOL000513	Gallic acid	0.01326848	0.36206897	15

**Table 2:** Therapeutic targets of Caulis Spatholobi on chronic myeloid leukemia.

**S. No.**	**Name**	**Betweenness Centrality**	**Closeness Centrality**	**Degree**
1	PTGS2	0.11949197	0.48540146	59
2	HSP90AA1	0.04264971	0.41736402	50
3	PTGS1	0.0762253	0.46941176	48
4	MAPK14	0.02118437	0.39426877	31
5	ESR1	0.01502596	0.38291747	31
6	PPARG	0.01343622	0.39194499	29
7	GSK3B	0.00983205	0.35123239	29
8	RXRA	0.01138571	0.35945946	28
9	NCOA2	0.00640128	0.32758621	28
10	NOS2	0.02073905	0.40466531	27
11	AR	0.01029261	0.37081784	25
12	RELA	0.02200394	0.3997996	24
13	CCNA2	0.00495812	0.33529412	23
14	ESR2	0.00353549	0.3325	23
15	CHEK1	0.00820708	0.3601083	22
16	CASP3	0.06686068	0.41134021	21
17	PIK3CG	0.01484474	0.38	21
18	MAPK1	0.00602156	0.36141304	21
19	TP53	0.00777637	0.37150838	19
20	ckd2	0.00567127	0.35498221	19
21	AKT1	0.00441683	0.35185185	19
22	ADRB2	0.00401754	0.32975207	19
23	MAPK3	0.00397377	0.34816754	19
24	TNF	0.00819603	0.37081784	18
25	NFKB1	0.00680947	0.34278351	18
26	PRSS1	0.0064382	0.36672794	18
27	FOS	0.0065101	0.36876155	17
28	CDKN1A	0.00615831	0.36808118	17
29	PDE3A	0.00776198	0.33529412	16
30	VEGFA	0.00563016	0.36538462	16
31	BAX	0.00445583	0.35752688	15
32	MAOB	0.01189871	0.328125	12
33	BCL2	0.00386875	0.31767516	12
34	NOS3	0.00880808	0.34816754	11
35	SRC	0.01503938	0.33473154	10
36	AKR1B1	0.02045636	0.35435169	9
37	ADH1C	0.00847086	0.30645161	9
38	cobT	0.00847086	0.30645161	9
39	CAT	0.00571203	0.34278351	8
40	ADH1B	0.00592652	0.30273141	7
41	PRSS3	0.00472278	0.30365297	6
42	IL4	0.00380535	0.34695652	6
43	ATP5F1B	0.00372972	0.33642496	6

**Table 3:** Inhibitory effect of alcohol extracts of Caulis Spatholobi on K562 cells.

**Concentration (μg/mL)**	**OD570**	**Inhibition (%)**
0	1.238 ± 0.158	0
15.62	1.060 ± 0.109	16.72
31.25	0.793 ± 0.089**	35.91
62.5	0.676 ± 0.061**	45.35
125	0.498 ± 0.023**	59.80
250	0.364 ± 0.042**	70.55
500	0.234 ± 0.020**	81.09

**Note:** Compared with control **p < 0.01. (x ± s, n = 4).

## Data Availability

The data used to support the findings of this study are available from the corresponding author upon request.

## LIST OF ABBREVIATIONS

Bax: Bcl-2-Associated X
Bcl-2: B-cell Lymphoma-2
Caspase-3: Cysteine Aspartate Protease3
CML: Chronic Myeloid Leukemia
TKI: Tyrosine Kinase Inhibitor
